# Nrf2 Knockout Affected the Ferroptosis Signaling Pathway against Cisplatin-Induced Hair Cell-Like HEI-OC1 Cell Death

**DOI:** 10.1155/2022/2210733

**Published:** 2022-07-01

**Authors:** Weilong Wang, Pengwei Ma, Wei Gao, Peiheng Lu, Xuerui Ding, Jiawei Chen, Hao Yuan, Lianjun Lu

**Affiliations:** Department of Otolaryngology Head & Neck Surgery, Tangdu Hospital, Fourth Military Medical University, Xi'an 710038, China

## Abstract

Cisplatin is a well-known and widely used anticancer drug with high therapeutic efficacy in solid tumors; however, side effects are common with its use. Because cisplatin can be retained in the cochlea, ototoxicity leading to hearing loss limits its clinical applications. Here, we report that Nrf2 knockout (KO) strongly increased cisplatin resistance in HEI-OC1 cells, which are immortalized cells from the murine organ of Corti. The underlying mechanism of this phenomenon was uncovered, and an important novel therapeutic target for combating cisplatin-induced hearing loss was identified. Preliminary investigations determined that Nrf2 KO markedly decreased TfR1 protein levels and increased GPX4 protein levels. Thus, ferroptosis may protect organisms from cisplatin-induced cell death. Furthermore, Nrf2 KO cells were resistant to the classical ferroptosis inducers RSL3 and erastin, providing solid evidence that Nrf2 KO inhibits ferroptosis and that knocking out Nrf2 may be a new clinical strategy to prevent cisplatin-induced hearing loss.

## 1. Introduction

With the development of society and medical technology, cancer has become easier to detect, but cancer treatment results in a substantial burden on all patients [1]. Since cisplatin was first discovered in the 1970s [2], it has been a very effective cancer chemotherapeutic in both children and adults [3]. However, cisplatin therapy is limited by severe side effects, including ototoxicity, nephrotoxicity, and neurotoxicity. One side effect is irreversible sensorineural hearing loss, which occurs in many cancer patients treated with cisplatin [4, 5] because cisplatin can accumulate in the cochlea [6]. Thus, the ototoxic side effects limit the clinical applications of cisplatin, and few methods have been developed to overcome this limitation.

Previous studies have shown that ototoxic reactive oxygen species (ROS) accumulation generated by cisplatin exposure induces severe cell death in cochlear hair cells and spiral ganglion neurons. Therefore, reducing free radicals might be an effective means to prevent cisplatin-induced hair cell loss. The most effective clinical drug currently under study to treat chemotherapy side effects is sodium thiosulfate [7–9].

In addition to ROS accumulation, necroptosis is also believed to participate in cisplatin-induced ototoxicity. Researchers used a specific inhibitor of necroptosis, Nec-1, to suppress cisplatin-induced cell death in HEI-OC1 cells, whereas a well-known apoptosis inhibitor, Z-VAD, did not inhibit cisplatin-induced cell death. These results suggested that necroptosis is involved in cisplatin-induced cell death [10].

Ferroptosis, an iron-dependent type of cell death that was identified in recent years, mainly involves lipid ROS-oxidizing membranes. Ferroptosis is involved in multiple signaling pathways and pathological conditions. Researchers have found that ferrostatin-1 (Ferr-1), a specific ferroptosis inhibitor, can effectively inhibit cisplatin-induced HEI-OC1 cell death. Moreover, inhibition of the ferroptosis signaling pathway protects cochlear hair cells from cisplatin-induced cell death. This result indicated that inhibition of the ferroptosis signaling pathway could reduce lipid peroxide radicals, protect mitochondrial function, and alleviate cisplatin-induced hearing loss [11]. In addition, the authors found that treatment with the ferroptosis inhibitor liproxstatin-1 (Lip-1) may be a new clinical intervention to prevent neomycin-induced hearing loss [12]. Furthermore, the combination of Ferr-1 and 3-methyladenine (3-MA) ameliorated asthma *in vivo* and *in vitro* by inhibiting ferroptosis [13].

The nuclear factor erythroid 2-related factor 2 (Nrf2) and antioxidant response element (ARE) signaling pathways protect against oxidative stress by inhibiting ROS accumulation. We observed that Nrf2 protein expression varied at different time points after cisplatin treatment and found that the Nrf2 protein level increased from 0.5–8 h followed by degradation of the protein from 16–24 h. In the early stage (0.5-8 h), Nrf2 accumulation may decrease ROS levels and increase cytoprotection. In the later stage (16-24 h), complete Nrf2 protein degradation may be an unusual signal. We found that Nrf2 gene knockout (KO) in HEI-OC1 cells led to resistance to cisplatin. Even when the cisplatin concentration was increased to 100 *μ*M, no cell death was observed; therefore, a cell line that is immune to cisplatin injury was constructed. However, elucidation of the underlying mechanisms these observations is urgently needed to identify more effective therapies to prevent cisplatin-induced ototoxicity.

## 2. Materials and Methods

### 2.1. Cell Culture and Nrf2 KO Cell Line Construction

In our experiments, we used HEI-OC1 cells constructed by the House Ear Institute from the organ of Corti, which is consistent with many previous inner ear studies [14–17]. This is an immortalized cochlear sensory epithelial cell line that expresses multiple hair cell markers. The HEI-OC1 cell line was cultured in DMEM (HyClone, SH30243, GE Healthcare Life Sciences) containing 10% fetal bovine serum (FBS) (Gibco, 10099, Life Technologies Australia Pty Ltd.) and 1% penicillin–streptomycin solution (HyClone, SH40003, GE Healthcare Life Sciences) at 37°C under 5% CO_2_ [18–20].

The PX459 plasmid designed by the Zhang laboratory was purchased from Addgene and digested by BbsI endonuclease. CRISPR/Cas9 combination sequences to target genomic sites in Nrf2 were determined by an online design tool from MIT (http://crispr.mit/edu/), Nrf2 sgRNA: GGAGTAGCTGGCGGATCCAC. Then, two synthesized single-stranded DNAs were annealed to form double-stranded DNA containing the BbsI cohesive end, and the PX459-Nrf2 plasmid was sequenced.

For transfection, HEI-OC1 cells were plated onto 60 mm plates, and 1 *μ*g of each plasmid was mixed with Polyplus transfection reagent (#01BIM0110a6, France) in jetOPTIMUS buffer according to the manufacturer's instructions. HEI-OC1 cells were treated with 5 *μ*g/mL puromycin for 72 h posttransfection and then digested and cultured in 100 mm plates. The cells were cultured for 7–14 days, and monoclonal cell clusters were obtained and analyzed by Western blotting.

### 2.2. Cisplatin Injection in CBA/J Mice

Eight-week-old male CBA/J mice purchased from the Jackson Laboratory were divided into two groups after passing a hearing test. In the control group, CBA/J mice were administered normal saline daily whereas the mice in the cisplatin treatment group were administered cisplatin (Tocris, 2251) diluted with normal saline (0.9% NaCl) by intraperitoneal injection at a dosage of 10 mg/kg. To prevent dehydration after cisplatin injection, we administered 1 mL of warm (37°C) normal saline by subcutaneous injection until the body weight recovered to that before cisplatin injection, and the cisplatin-treated mice were placed on the heating pad at all times. Auditory brainstem response (ABR) threshold shifts were measured after the recovery period of the second cycle.

### 2.3. Cell Viability Analysis

Cell Counting Kit-8 (CCK-8) (Dojindo, Japan) reagent was used to examine cell viability according to the manufacturer's instructions. On the first day, we seeded HEI-OC1 cells at a concentration of 6000 cells/well in 96-well plates with 5 replicates for overnight culture. The following day, we pretreated the cells with cisplatin for another 24 h. On the third day, we exchanged the culture medium with 90 *μ*L of fresh DMEM plus 10 *μ*L of CCK-8 reagent, incubated the cells for 1–2 h at 37°C under 5% CO_2_, and used a plate reader (BioTek, Epoch) to measure absorbance at 450 nm. All experiments were repeated at least 3 times.

### 2.4. Lactate Dehydrogenase (LDH) Release Assay

LDH is a marker found in the cytoplasm. When the cell membrane is broken, this molecule is released from the cytoplasm of the cell into the culture medium. The amount of LDH leaked was determined using an LDH cytotoxicity detection kit (CytoTox 96 Non-Radioactive Cytotoxicity Assay kit (Promega)). Approximately 6000 HEI-OC1 and Nrf2 KO cells/well were cultivated separately in 96-well plates overnight and then treated with or without cisplatin. According to the manufacturer's instructions, 100 *μ*L of fresh reaction solution was added to each well for 30 min of incubation at room temperature (RT) after the different treatments, and a microplate reader (BioTek, Epoch) was used to estimate LDH release by measuring the absorbance at 492 nm. The classical ferroptosis inducers 1S,3R-RSL3 (#SML2234) and erastin (#E7781) were purchased from Sigma.

### 2.5. Flow Cytometry

Apoptosis analysis was carried out with a flow cytometry assay using an Annexin V-FITC/PI kit (BD Biosciences Pharmingen, 556547). HEI-OC1 cells and Nrf2 KO cells were treated with or without cisplatin for 24 h, collected by centrifugation at 1000 rpm for 5 min, washed twice with cold PBS, and resuspended in 1X binding buffer at a concentration of 1 × 10^6^ cells/mL. Annexin V-FITC and propidium iodide were added, gently mixed with the cells, and incubated for 15 min at RT in the dark. The stained cells were analyzed with a Beckman flow cytometer as soon as possible after incubation, and the data were processed using FlowJo software. All experiments were repeated three times.

### 2.6. TUNEL Assay

To evaluate DNA strand breaks in cells undergoing apoptosis, a TUNEL Assay Kit (Roche, Indianapolis, IN, USA) was used to label DNA strand breaks with TdT, which catalyzes the polymerization of labeled nucleotides into free 3′-OH DNA ends. After HEI-OC1 or Nrf2 KO cells were treated with or without 25 *μ*M cisplatin for 24 h, all samples were washed with PBS 3 times, fixed in 4% paraformaldehyde (PFA) for 20 min at RT, and treated with 0.1% Triton X-100 for 15 min. Then, 100 *μ*L of TUNEL reaction mixture was added to each sample for 60 min of incubation at 37°C, and DAPI-labeled cell nuclei were observed.

### 2.7. Western Blot Analysis

HEI-OC1 and Nrf2 KO cells were lysed with RIPA lysis buffer (Beyotime Biotech Inc., P0013B) plus protease inhibitor cocktail tablets (Roche, 04693159001) and then total protein quantified with a BCA Protein Assay Kit (Beyotime Biotech Inc., P0012S), and 5X SDS–PAGE protein loading buffer (Beyotime Biotech Inc., P0015 L) was added. The lysed cells were suspended in a water bath kettle at 100°C for 5 min and then centrifuged at 4°C and 12000 rpm for 15 min. Samples were separated via 10% SDS–PAGE and transferred to 0.45 *μ*m pore diameter PVDF membranes (Merck Millipore Ltd., IPVH00010) when the target proteins weighed more than 25 kD. Samples were separated via 12% SDS–PAGE and transferred to 0.2 *μ*m pore diameter PVDF membranes for target proteins weighing less than 25 kD (Merck Millipore Ltd., ISEQ00010). Then, the PVDF membranes were blocked with skim milk (BD, America, 232100) in TBST buffer at RT for 1 h. The membranes were probed with Nrf2 (N2C2, GeneTex, Inc.), KEAP1 (#8047, Cell Signaling Technology, Inc.), p53 (#32532, Cell Signaling Technology, Inc.), CTR1 (#13086, Cell Signaling Technology, Inc.), GPX4 (ab125066, Abcam, Inc.), and TfR1 (#13-6890, Invitrogen) antibodies at a 1 : 1000 dilution. The samples were next incubated with anti-GAPDH antibody (GTX100118, 1 : 4000, GeneTex, Inc.) overnight at 4°C. The following day, the PVDF membranes were washed with TBST 4 times for 15 min each time and then incubated with HRP-conjugated secondary antibodies at a 1 : 2000 dilution (Cell Signaling Technology, Inc.) for 1 h. Protein bands were detected using a chemiluminescence apparatus (Vilber Fusion Solo S, France). The expression of all proteins was normalized to GAPDH protein expression, and the assays were repeated at least 3 times.

### 2.8. RNA Extraction and Quantitative RT–PCR

Total RNA was isolated using TRIzol (Invitrogen) according to the manufacturer's protocol. One microgram of total RNA was reverse transcribed with 5X PrimeScript RT Master Mix (Takara, Japan) following the manufacturer's protocol. Quantitative PCR was performed using a CFX Connect Real-Time PCR System (Bio–Rad) according to a standard protocol. The quantitative RT–PCR primers used are as follows: actin forward, GTCCCTCACCCTCCCAAAAG, actin reverse, GCTGCCTCAACACCTCAACCC; TfR1 forward, TGGAG ACTACTTCCGTGCTAC, TfR1 reverse, TCCACTAAAGCTGAGAGGGTG; and GPX4 forward, GTTTCGTGTGCATCGTCACC, GPX4 reverse GGGCATCGTCCCCATTTACA.

### 2.9. FerroOrange Stain

FerroOrange is a novel fluorescent probe that enables live-cell fluorescence imaging of intracellular Fe^2+^ (Dojindo Laboratories, F374, Japan). A total of 2 × 10^5^ HEI-OC1 and Nrf2 KO cells were seeded separately on a dish for fluorescence imaging and cultured overnight in a 37°C incubator equilibrated with 5% CO_2_. The following day, the HEI-OC1 and Nrf2 KO cells were treated with or without 25 *μ*M cisplatin. After 24 h of treatment, the supernatant was discarded, and the cells were washed with HBSS two times. FerroOrange working solution (1 *μ*mol/L) was added to the cells for 10 min of incubation in a 37°C incubator equilibrated with CO_2_. Finally, the cells were observed under a fluorescence microscope (Olympus FV1000). All images are representative of three independent experiments.

### 2.10. Liperfluo Staining

Liperfluo, a Spy-LHP analog, is used for lipid peroxide detection. Although the oxidized form of Liperfluo is almost nonfluorescent in aqueous solutions, it emits fluorescence in lipophilic sites, such as in cell membranes; therefore, it can be easily applied to image lipid peroxides by fluorescence microscopy (Dojindo Laboratories, L248, Japan). The 10 *μ*mol/L Liperfluo working solution was added to the cells for 30 min of incubation in a 37°C incubator equilibrated with CO_2_ and then treated with DAPI (1 : 1000, Roche) for 10 min at RT. The cells were observed under a fluorescence microscope. All images are representative of three independent experiments.

### 2.11. MitoSOX Red Mitochondrial Superoxide Indicator

MitoSOX™ Red reagent is live-cell permeant that rapidly and selectively targets the mitochondria. Once in the mitochondria, the MitoSOX™ Red reagent is oxidized by superoxide to exhibit red fluorescence. MitoSOX™ reagent working solution (5 *μ*mol/L) was added to cells for 10 min of incubation in a 37°C incubator equilibrated with CO_2_. The cells were observed under a fluorescence microscope (Olympus FV1000). All images are representative of three independent experiments.

### 2.12. Statistical Analyses

Statistical analyses were performed with Prism software (GraphPad Software, La Jolla, CA). One-way ANOVA followed by a Newman–Keuls multiple comparisons test was used when comparing more than two groups. A two-sample Student's *t* test was used when only two conditions were compared. ns represents no significant difference, ∗ represents *p* < 0.05, ∗∗ represents *p* < 0.01, and ∗∗∗ represents *p* < 0.001.

## 3. Results

### 3.1. Cisplatin-Induced Hair Cell Death in the Mouse Cochlea

**Figure 1:**
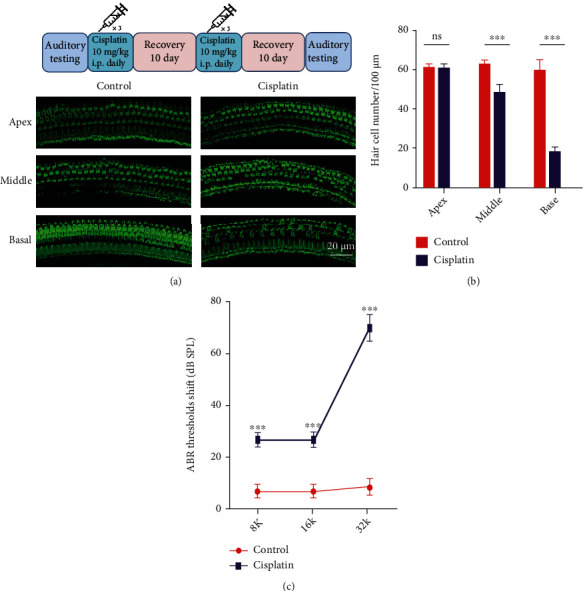
Cisplatin-induced cell death in the mouse cochlea. (a, top) Mode of cisplatin administration to CBA/J mice. (a, bottom) Phalloidin-labeled hair cells of the organ of Corti. (b) Hair cell quantification and statistical analysis. (c) Statistical analysis of the ABR threshold shift. ∗*p* < 0.05, ∗∗*p* < 0.01, and ∗∗∗*p* < 0.001. Scale bar, 20 *μ*m.

Cisplatin at a concentration of 10 mg/kg was administered to 8-week-old male CBA/J mice for 3 days, and then, the mice were allowed to recover to their normal weight for 10 days. This procedure was repeated for another cycle. After the second recovery period, the auditory threshold of each mouse was tested, the mice were sacrificed, and fluorescent phalloidin was used to label the hair cells. From the results, we found that cisplatin induced substantial hair cell loss, especially in the basal turn (Figures 1(a) and 1(b)). ABR testing indicated that the cisplatin-induced ABR threshold increased at all frequencies, and the most serious increase was observed in the high-frequency region (Figure 1(c)).

### 3.2. Cisplatin-Induced Hair Cell-Like HEI-OC1 Cell Death

**Figure 2:**
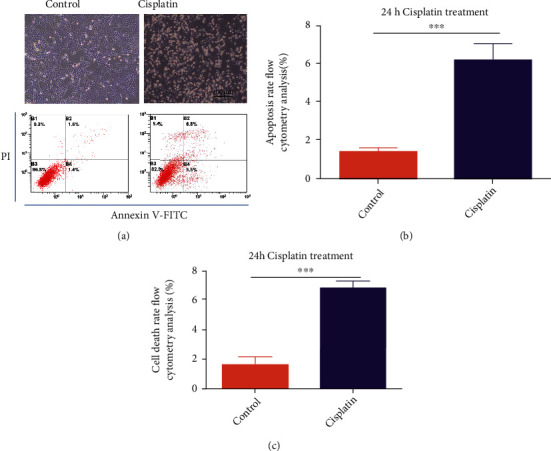
Cisplatin-induced HEI-OC1 cell death. (a) Light field and flow cytometry analysis after cells were treated with 25 *μ*M cisplatin for 24 h. (b) Number of early apoptotic cells in A. (c) Number of dead cells in A. ∗*p* < 0.05, ∗∗*p* < 0.01, and ∗∗∗*p* < 0.001. Scale bar, 100 *μ*m.

The hair cell-like HEI-OC1 cells used in this study are immortalized cells from the murine organ of Corti. Consistent with many previous inner ear studies, we treated HEI-OC1 cells with 25 *μ*M cisplatin for 24 h and found that many of the cells had died; the flow cytometry results were consistent with the above finding (Figure 2(a)). Statistical analyses showed that the numbers of early apoptotic cells and dead cells significantly increased in the cisplatin treatment group (Figures 2(b) and 2(c)).

### 3.3. Nrf2 May Regulate Cisplatin-Induced HEI-OC1 Cell Death

**Figure 3:**
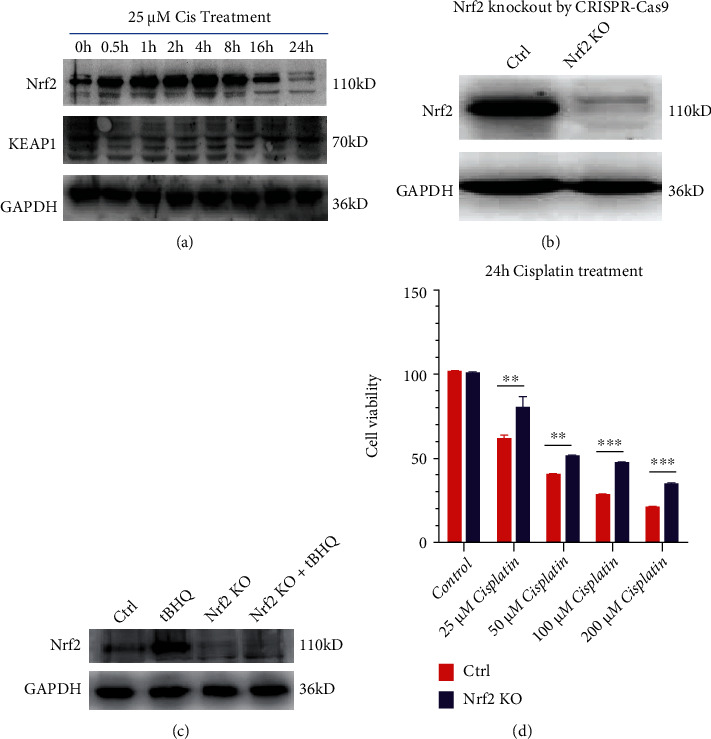
Nrf2 participates in cisplatin-induced HEI-OC1 cell death. (a) Western blots of Nrf2, KEAP1, and GAPDH after cisplatin treatment for different lengths of time. (b) Western blot confirming Nrf2 KO in the desired cell line. (c) Control and Nrf2 KO cells were treated with 25 *μ*M tBHQ for 24 h. (d) Control and Nrf2 KO cells treated with varying concentrations of cisplatin for 24 h were analyzed by CCK-8 assays. ∗*p* < 0.05, ∗∗*p* < 0.01, and ∗∗∗*p* < 0.001.

To explore the underlying mechanism of cisplatin-induced HEI-OC1 cell death, we treated HEI-OC1 cells with 25 *μ*M cisplatin for 0.5 h, 1 h, 2 h, 4 h, 8 h, 16 h, and 24 h; and Nrf2 and KEAP1 protein expression was observed at each time point. Cisplatin treatment sharply increased the Nrf2 protein levels from 0.5–8 h while Nrf2 was degraded from 16–24 h. Changes in the protein expression of KEAP1, a negative regulator of Nrf2, were also observed (Figure 3(a)). Based on the above results, we hypothesized that the Nrf2 signaling pathways may regulate HEI-OC1 cell survival and death. To determine whether Nrf2 KO HEI-OC1 cells were sensitive or resistant to cisplatin, we knocked out the Nrf2 gene by CRISPR–Cas9 technology and assessed the cells with Western blot assays (Figure 3(b)). To ensure that Nrf2 KO was complete, we added the Nrf2 activator tBHQ to the Nrf2 KO cell line and found that Nrf2 KO was satisfactory (Figure 3(c)). Then, the HEI-OC1 and Nrf2 KO cell lines were exposed to different concentrations of cisplatin to analyze cell viability by performing CCK-8 assays. As shown in Figure 3(d), exposure to 25, 50, 100, and 200 *μ*M cisplatin for 24 h resulted in a dose-dependent decrease in cell viability, confirming the ototoxic effect of cisplatin on HEI-OC1 cells. Notably, ototoxicity was clearly attenuated in Nrf2 KO cells.

### 3.4. Nrf2 KO Substantially Increases Cisplatin Resistance in HEI-OC1 Cells

**Figure 4:**
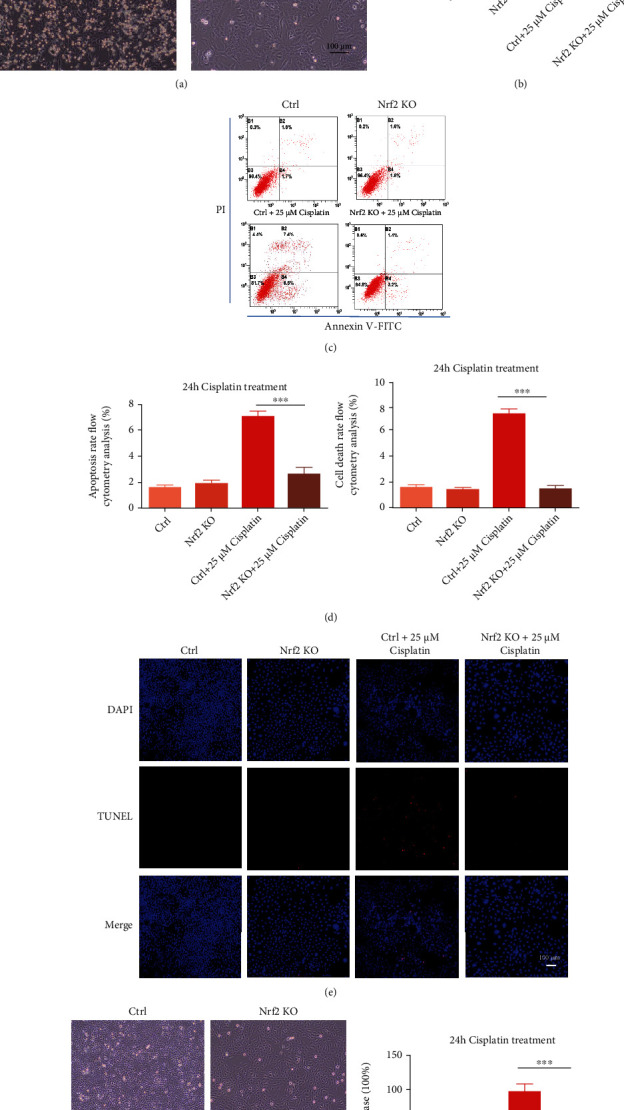
Nrf2 KO strongly increases cisplatin resistance in HEI-OC1 cells. (a) Images showing control and Nrf2 KO cells after 25 *μ*M cisplatin treatment for 24 h in a light field. (b) LDH assays of the cells in A. (c) Flow cytometry analysis of cell apoptosis of the cells in A. (d) Number of early apoptotic cells in C. (e) DAPI and TUNEL double labeling of apoptotic cells after treatment with 25 *μ*M cisplatin. (f) Image showing control and Nrf2 KO cells after 100 *μ*M cisplatin treatment for 24 h in a light field. (g) LDH assays of the cells in F. ns: no significant difference, ∗*p* < 0.05, ∗∗*p* < 0.01, and ∗∗∗*p* < 0.001. Scale bar, 100 *μ*m.

According to the above cell viability data, we selected 25 *μ*M cisplatin as an appropriate concentration for subsequent studies. The HEI-OC1 and Nrf2 KO cell lines remained untreated or were treated with 25 *μ*M cisplatin for 24 h and then photographed in a light field. In the images, there were many bright dead cells in the HEI-OC1 group but not in the Nrf2 KO group (Figure 4(a)). We found notable cisplatin resistance in Nrf2 KO cells compared with HEI-OC1 cells. To further verify that the Nrf2 KO cell line was resistant to cisplatin, we performed flow cytometry, and the results showed substantially fewer apoptotic cells in the Nrf2 KO cells than in the HEI-OC1 cells after exposure to cisplatin (Figures 4(c) and 4(d)). Moreover, after treatment with 25 *μ*M cisplatin for 24 h, no TUNEL-positive Nrf2 KO cells were observed. In contrast, in the HEI-OC1 group, the number of TUNEL-positive cells distinctly increased (Figure 4(e)). To confirm cisplatin resistance in the Nrf2 KO cell line, we used increasing concentrations of cisplatin, up to 100 *μ*M, to evaluate cytotoxicity. When the cells were treated with very high concentrations of cisplatin, most of the HEI-OC1 cells died, while few Nrf2 KO cells died (Figure 4(f)). The LDH assay results were in accordance with this finding (Figure 4(g)).

### 3.5. Cisplatin Resistance in Nrf2 KO Cells Is Regulated by the Ferroptosis Signaling Pathway

Previous literature has demonstrated that 18-month-old Nrf2 KO mice displayed remarkably improved motor coordination, which may result from reduced ROS levels and decreased apoptosis in the neurons in the substantia nigra (SN). The regulation of Nrf2 KO on brain iron metabolism was found to be mediated by decreasing the ferroportin 1 (FPN1) levels in brain microvascular endothelial cells, thus hindering the process of iron entry into the brain [21]. Ferroptosis is a form of regulated cell death that is characterized by the iron-dependent accumulation of lipid hydroperoxides. Sensitivity to ferroptosis is tightly linked to numerous biological processes, including iron and polyunsaturated fatty acid metabolism and glutathione biosynthesis [22].

**Figure 5:**
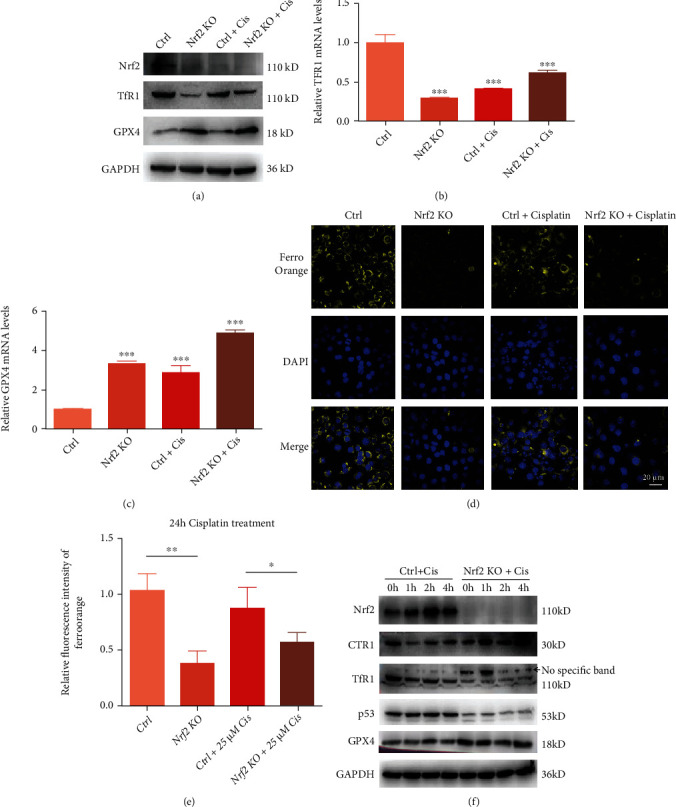
Nrf2 KO affects ferroptosis signaling pathway-induced cisplatin resistance. (a) Western blots of Nrf2, TfR1, and GPX4 protein expressions in control and Nrf2 KO cells with or without 25 *μ*M cisplatin treatment for 24h. (b) RT -PCR verification of TfR1 mRNA expression levels in control and Nrf2 KO cells with or without 25 *μ*M cisplatin treatment for 24h. (c) RT -PCR verification of GPX4 mRNA expression levels in control and Nrf2 KO cells with or without 25 *μ*M cisplatin treatment for 24h. (d) FerroOrange indicated the intracellular Fe^2+^ content after treatment with 25 *μ*M cisplatin for 24h. (e) Relative fluorescence intensity of FerroOrange for the cells in (d). (f) Western blots of Nrf2, CTR1, TfR1, p53, and GPX4 protein expressions in control and Nrf2 KO cells treated with 25 *μ*M cisplatin for different lengths of time. ∗*p* < 0:05, ∗∗*p* < 0:01, and ∗∗∗*p* < 0:001. Scale bar, 20 *μ*m.

Herein, we found that Nrf2 KO significantly decreased the expression level of transferrin receptor 1 (TfR1), thus hindering the process of iron endosomal uptake into cells and affecting iron metabolism. In addition, Nrf2 KO remarkably improved the GPX4-glutathione axis (Figure 5(a)). RT–PCR was used to detect the mRNA levels of TfR1 and GPX4, and the results were consistent with the protein detection results (Figures 5(b)–5(c)). FerroOrange is a novel fluorescent probe that enables live-cell fluorescence imaging of intracellular ferrous ions (Fe^2+^). The images acquired showed that the contents of ferrous ions decreased remarkably in Nrf2 KO cells, a result that is consistent with the reduction in TfR1 levels (Figures 5(d) and 5(e)). Furthermore, we determined the Nrf2 protein levels in control and Nrf2 KO cells in response to cisplatin treatment at the early stage. The Nrf2 protein was activated in the control group but not detected in the Nrf2 KO group. To exclude Nrf2 KO-affected cisplatin entry into cells, CTR1, a high-affinity copper uptake protein that assists in maintaining copper homeostasis, also mediates the uptake of the anticancer drug cisplatin. The results showed that Nrf2 KO did not decrease CTR1 protein levels, indicating that cisplatin can enter Nrf2 KO cells as easily as control cells. The TfR1 protein level was significantly decreased in Nrf2 KO cells after exposure to cisplatin for different lengths of time. Moreover, GPX4 protein expression was significantly increased in Nrf2 KO cells, which increased the cell reduction system. Notably, p53 degradation may suppress the apoptosis pathway and promote cell survival. Taken together, these results suggest that Nrf2 KO may inhibit the ferroptosis signaling pathway by degrading TfR1 and accumulating GPX4 to increase cisplatin resistance.

### 3.6. Nrf2 KO Alleviates Mitochondrial ROS and Lipid Peroxide Levels after Cisplatin Treatment

**Figure 6:**
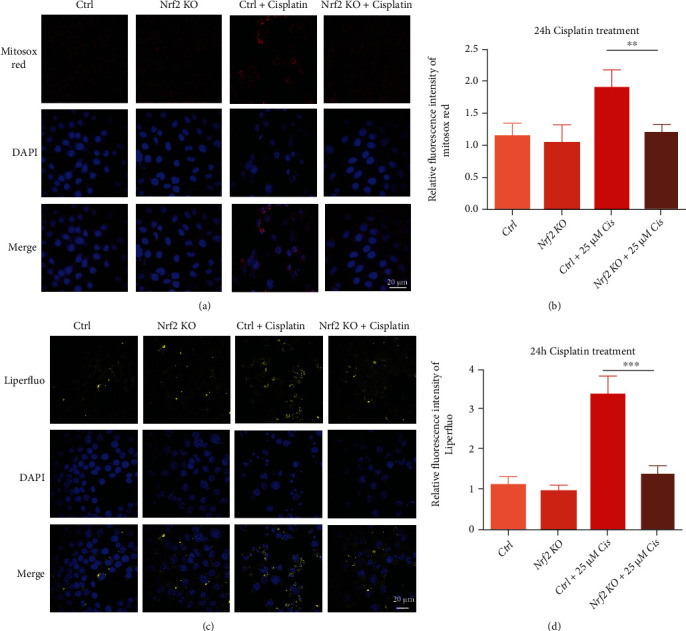
Nrf2 KO alleviated mitochondrial ROS and lipid peroxide levels after cisplatin treatment. (a) MitoSOX Red staining indicated mitochondrial ROS accumulation in cells after treatment with 25 *μ*M cisplatin for 24 h. (b) Relative fluorescence intensity of MitoSOX Red in the cells in A. (c) Liperfluo indicated the lipid peroxide levels in cells after treatment with 25 *μ*M cisplatin for 24 h. (d) Relative fluorescence intensity of Liperfluo in the cells in C. ∗*p* < 0.05, ∗∗*p* < 0.01, and ∗∗∗*p* < 0.001. Scale bar, 20 *μ*m.

MitoSOX Red is a live-cell permeant dye that selectively targets the mitochondria. After entry into the mitochondria, this reagent is oxidized by superoxide and exhibits red fluorescence. The images in Figure 6(a) show that the fluorescence from MitoSOX Red was markedly decreased in Nrf2 KO cells after treatment with 25 *μ*M cisplatin compared with the control group cells, which might account for the reduction in Nrf2 KO cell death. Liperfluo is used for lipid peroxide detection, as it emits fluorescence in lipophilic sites such as in cell membranes, which are made up of peroxidized phospholipids that are the key drivers of ferroptotic death. Therefore, Liperfluo can be easily applied for lipid peroxide imaging by fluorescence microscopy. As shown in Figure 6(c), compared to undamaged control cells, Liperfluo signals were significantly increased after cells were treated with 25 *μ*M cisplatin, but these increases were significantly inhibited in the Nrf2 KO cell line. Nrf2 KO significantly reduced lipid peroxidation and eliminated the lipid peroxides, thus controlling the abundance of key phospholipid substrates involved in the regulation of ferroptosis.

### 3.7. Nrf2 KO Cells Are Resistant to the Classical Ferroptosis Inducer RSL3

Ferroptosis is a form of regulated cell death driven by iron-dependent lipid peroxidation. Peroxidized phospholipids, which compose the lipid bilayers that make up cellular membranes, are the key driver of ferroptotic death. Regulation of ferroptosis involves controlling the abundance of key phospholipid substrates, the factors that drive their peroxidation, and the factors that eliminate these lipid peroxides. Ferroptosis inducers, such as RSL3, inhibit GPX4 function and induce lipid peroxidation [23].

**Figure 7:**
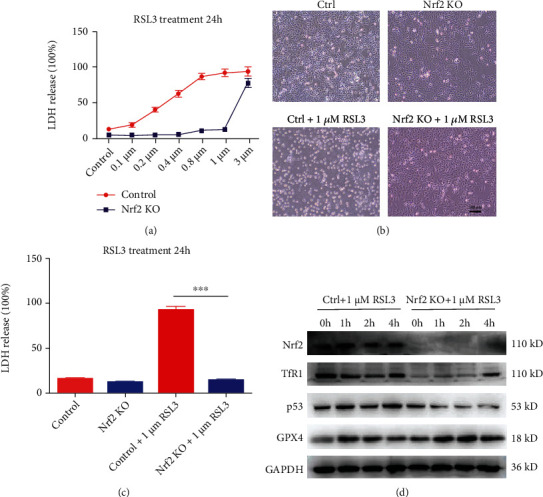
The Nrf2 KO cell line was resistant to the ferroptosis inducer RSL3. (a) LDH release levels in control and Nrf2 KO cells treated with different concentrations of RSL3 for 24 h. (b) Image showing control and Nrf2 KO cells after 1 *μ*M RSL3 treatment for 24 h in a light field. (c) LDH release levels in B. (d) Western blots of Nrf2, CTR1, TfR1, p53, and GPX4 protein expression in control and Nrf2 KO cells treated with 1 *μ*M RSL3 at different time points. ∗*p* < 0.05, ∗∗*p* < 0.01, and ∗∗∗*p* < 0.001. Scale bar, 100 *μ*m.

Control and Nrf2 KO cells were exposed to 0.1, 0.2, 0.4, 0.8, 1, and 3 *μ*M RSL3 for 24 h. In the control group of cells, RSL-3 treatment resulted in a dose-dependent increase in LDH release. In the Nrf2 KO group, LDH release did not change after treatment with 0.1 to 1 *μ*M RSL3 for 24 h but remarkably improved after treatment with 3 *μ*M RSL3 (Figure 7(a)). According to the LDH release data, we selected 1 *μ*M RSL3 as an appropriate concentration for subsequent studies. Control and Nrf2 KO cells remained untreated or were treated with 1 *μ*M RSL3 for 24 h and were then photographed in a light field. In the acquired images, there were many bright dead cells in the control group but not in the Nrf2 KO group (Figure 7(b)). The LDH assay confirmed the above result (Figure 7(c)). In addition, we evaluated the expression levels of Nrf2, TfR1, p53, and GPX4 in the control and Nrf2 KO groups treated with 1 *μ*M RSL3 for 1, 2, and 4 h by Western blotting. The Nrf2 protein was activated in the control group of cells and not detected in the Nrf2 KO group. The control group expressed abundant TfR1, while the Nrf2 KO group showed markedly decreased TfR1 contents. The results also showed that p53 protein content was significantly decreased in the Nrf2 KO group compared with the control group, while GPX4 expression was increased in the Nrf2 KO group compared with the control group. These results indicated that the cells in the Nrf2 KO group had decreased TfR1 protein expression, decreased iron imported to the intracellular cell membrane, and increased GPX4 protein levels to inhibit lipid peroxidation. From these results, we concluded that the Nrf2 KO cell line was resistant to the classical ferroptosis inducer RSL3.

### 3.8. Nrf2 KO Cells Are Resistant to the Classical Ferroptosis Inducer Erastin

**Figure 8:**
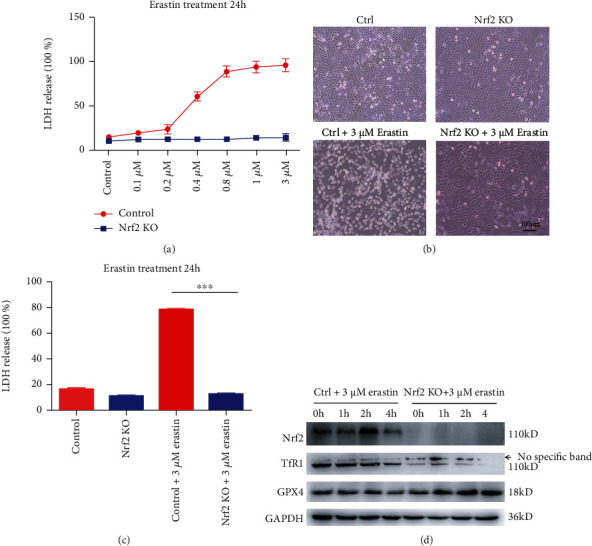
Nrf2 KO cells were resistant to the ferroptosis inducer erastin. (a) LDH release levels in control and Nrf2 KO cells treated with different concentrations of erastin for 24 h. (b) Image showing control and Nrf2 KO cells after 3 *μ*M erastin treatment for 24 h in a light field. (c) LDH release levels in B. (d) Western blots of Nrf2, CTR1, TfR1, and GPX4 protein expression in control and Nrf2 KO cells treated with 3 *μ*M erastin at different time points. ∗*p* < 0.05, ∗∗*p* < 0.01, and ∗∗∗*p* < 0.001. Scale bar, 100 *μ*m.

Erastin, like glutamate, inhibits cystine uptake by the cystine/glutamate antiporter (system *X*_*c*_^−^), which exchanges extracellular cystine for intracellular glutamate [24]. Unlike RSL3, 3 *μ*M erastin did not increase LDH release in Nrf2 KO cells (Figure 8(a)). Based on the LDH release data, we selected 3 *μ*M erastin as an appropriate concentration for subsequent studies (Figures 8(b)–8(d)). All of these results indicated that Nrf2 KO inhibited iron uptake and GSH promotion to promote cell survival through the TfR1 and GPX4 signaling pathways.

## 4. Discussion

Cells subjected to external stress, such as H_2_O_2_, irradiation, and cisplatin, can generate ROS, which can cause DNA damage, trigger stress responses, and activate the DNA repair pathway to maintain genomic stability [25]. ROS promote mitochondrial dysfunction, genomic instability, and cancer development [26]; however, there are many small molecules that regulate reactive oxygen species homeostasis and show efficacy in cancer therapy [27]. Under normal circumstances, the transcription factor Nrf2 and its repressor protein KEAP1 are tightly bound to inhibit Nrf2 activity, but in response to cellular stress, the Nrf2-KEAP1 interaction is disrupted to stabilize the Nrf2 protein level and increase the transcription of Nrf2 downstream genes, including HO-1, NQO1, SRXN1, HMOX1, and SLC7A11; these genes directly or indirectly regulate redox balance [28]. KEAP1, the most effective negative regulator that directly binds to Nrf2, has more than 20 free sulfhydryl (-SH) groups in its protein component. The sulfhydryl groups of cysteine residues can reduce free oxides, including ROS and reactive nitrogen species (RNS), resulting in a conformational change in KEAP1 [29–32], thereby rescuing Nrf2 from proteasomal degradation and resulting in its accumulation and increased transcriptional activity [33].

Whether Nrf2 activation has positive or negative effects is unclear. It has been reported in the literature that common oncogenes, such as KRAS, BRAF, and MYC, all increase Nrf2 activity, which alleviates ROS accumulation and protects cells from oxidative damage [34]. Thus, a more favorable microenvironment for tumor cell survival is generated by Nrf2 activation. A previous study examined 304 lung cancer specimens [35], and abnormally high expression levels of Nrf2 were found in nonsmall-cell lung cancer samples by immunohistochemistry. Furthermore, P62 increases Nrf2 activity by suppressing KEAP1 expression and enhancing subsequent Nrf2 nuclear accumulation [36]. ARF, another important negative regulator of Nrf2, inhibits Nrf2 transcriptional activity. Therefore, ARF inhibition induces Nrf2 accumulation in response to oxidative stress and promotes cancer cell survival, while ARF activation sensitizes cells to death [37]. Nrf2 regulates downstream genes, such as HO-1, NQO1, SRXN1, HMOX1, and SLC7A11, functioning to detoxify oxidative species and the glutathione reduction system [38].

Nrf2 activation protects organisms by directly or indirectly regulating the redox balance through the Nrf2 downstream genes. In contrast to the traditional view, it was recently reported that mice with homozygous deletion of Nrf2 increased bone marrow stromal and hematopoietic progenitor cell radio-resistance, which is one form of oxidative stress [39]. In addition to evidence of irradiation resistance acquired from the abdominal small intestines of Nrf2 KO mice, Nrf2 KO prompts the proliferation and differentiation of Lgr5^+^ intestinal stem cells and the activation of NF-*κ*B [40]. The pharmacogenomics of cisplatin-induced ototoxicity in individual variability research has shown that the rs6721961 A-allele in promoter sequences results in an approximately 40% reduction in Nrf2 expression, which could therefore protect against cisplatin-induced hearing loss in cancer patients, and this increase in cellular sensitivity to cisplatin is the opposite of that which might have been expected [41]. Therefore, Nrf2 activation or inhibition has been inadequately framed, determining whether to use drugs to stimulate or inhibit the Nrf2 pathway may depend on the specific context.

In our study, cisplatin-induced cell death was observed in both the mouse cochlea and HEI-OC1 cells (Figures 1 and 2); thus, reducing hair cell loss in the cochlea is important for patients treated with cisplatin. Uncovering the underlying mechanism and identifying a novel therapeutic target has clinical significance. We observed that Nrf2 protein expression varied with different lengths of cisplatin treatment and may affect cell fate decisions.

Here, we report that Nrf2 KO inhibits cisplatin-induced HEI-OC1 cell death (Figures 3 and 4). As an almost universal rule, cisplatin can induce ROS and lipid peroxide radical accumulation. Therefore, it is logical to hypothesize that when the Nrf2 gene is knocked out, the production of free radicals induced by cisplatin increases, which expedites DNA damage and lipid peroxidation generation in the cell membrane through apoptosis and ferroptosis. Ferroptosis is a regulated form of cell death characterized by the iron-dependent incorporation of polyunsaturated fatty acids into cellular membranes. Emerging evidence suggests that ferroptosis is genetically, biochemically, and morphologically distinct from other cell death modalities.

Labile iron is imported through TfR1 [42] and stored in ferritin. We observed a dramatic decrease in the expression of TfR1 in Nrf2 KO cells compared with control cells (Figure 5(a)). Consistent with this finding, the content of FerroOrange-labeled intracellular ferrous ions (Fe^2+^) markedly decreased in the Nrf2 KO cell line (Figure 5(d)).

GPX4 is a pivotal enzyme that uses glutathione as an essential cofactor for its activity to reduce lipid hydroperoxides within biological membranes [43]. Studies have reported that dramatic degeneration of motor neurons occurs in the spinal cord upon conditional ablation of GPX4 in neurons of adult mice, resulting in the progression of paralysis [44]. Moreover, GPX4 -/- mice died shortly after birth and presented extensive hepatocyte degeneration, and GPX4 -/- livers manifested the upregulation of Nrf2 response genes. Remarkably, GPX4 -/- pups born to mothers fed a vitamin E-enriched diet survived, yet this protection was reversible, as subsequent vitamin E deprivation caused the death of the GPX4-deficient mice 4 weeks thereafter [45]. Loss of GPX4 activity and subsequent accumulation of lipid hydroperoxides lead to ferroptosis execution. In contrast, augmented GPX4 activity may eliminate lipid hydroperoxides and restrain ferroptosis [23]. In our study, GPX4 protein expression was markedly increased in the Nrf2 KO cell line with or without cisplatin exposure (Figure 5(a)), and the GPX4 mRNA transcriptional level also increased, consistent with its protein accumulation (Figure 5(c)). MitoSOX Red, which marks mitochondrial ROS, showed markedly decreased fluorescence in Nrf2 KO cells line compared with control cells. Liperfluo is used for the statin peroxidation of phospholipids in cell membranes. Liperfluo signals increased significantly after cisplatin treatment, but these increases were significantly inhibited in Nrf2 KO cells, as the abundance of ROS and phospholipid substrates involved in the regulation of ferroptosis were controlled (Figure 6).

To demonstrate that the Nrf2 KO cell line was resistant to cisplatin due to inhibition of the ferroptosis pathway, the ferroptosis inducers RSL3 [46] and erastin [47], which were first discovered using high-throughput screening of small molecule libraries, were used in this study. The cell death phenotype induced by RSL3 and erastin was distinct from that caused by other lethal compounds that bring about apoptosis and necroptosis. A previous paper demonstrated that RSL3 inhibits GPX4 enzymatic activity and induces ferroptosis. Erastin treatment abolished the import of radiolabeled cystine, a substrate for the system *X*_*c*_^−^ antiporter, confirming that erastin inhibits system *X*_*c*_^−^ and depletes GSH. In our study, compared with the control, Nrf2 KO cells were very resistant to the ferroptosis inducers RSL3 (Figures 7(a)–7(c)) and erastin (Figures 8(a)–8(c)), the Western blot assay showed that the TfR1 protein level was substantially decreased, and the GPX4 protein level was remarkably increased in the Nrf2 KO cell line. Iron acts as a catalyst for Fenton chemistry for the conversion of peroxides into free radicals, such as hydroxyl and hydroperoxyl radicals, and accounts for the lethal effects of ferroptosis, which is inhibited by iron transporters. The TfR1 level was found to decrease, and GPX4 showed increased cell function to eliminate lipid hydroperoxides, thereby inhibiting ferroptosis and maintaining cell viability (Figures 7(d) and 8(d)).

Overall, cisplatin-induced cell death was observed in both the mouse cochlea and HEI-OC1 cells. Nrf2 activity is desirable for HEI-OC1 cells during the early stages of cisplatin treatment when the host seeks to control ROS accumulation, but Nrf2 activity is undesirable during the later stages, when cell fate decisions may mediate cell survival. Herein, we report that in contrast to previous results, Nrf2 KO substantially inhibited cisplatin-induced HEI-OC1 cell death. We preliminarily investigated this phenomenon and found that Nrf2 KO inhibited ferroptosis by remarkably decreasing TfR1 protein levels and increasing GPX4 protein levels. The Nrf2 KO cell line was resistant to the classical ferroptosis inducers RSL3 and erastin, providing solid evidence that Nrf2 KO inhibits ferroptosis and may be a new clinical strategy to prevent cisplatin-induced hearing loss. Uncovering the underlying mechanism of this phenomenon may allow for the development of a novel therapeutic strategy to combat cisplatin-induced hearing loss.

## Data Availability

The original contributions presented in the study are included in the article/Supplementary Material, and further inquiries can be directed to the corresponding author.
